# Amyloid cranial polyneuropathy: A rare neurological presentation of immunoglobulin light‐chain amyloidosis

**DOI:** 10.1002/agm2.12148

**Published:** 2021-02-05

**Authors:** Abhinav Agarwal, Benny Paul Wilson, Prasad Kuruvilla Mathews, Surekha Viggeswarpu, Gopinath Kango Gopal

**Affiliations:** ^1^ Department of Internal Medicine Christian Medical College and Hospital Vellore Vellore India; ^2^ Department of Geriatrics Christian Medical College and Hospital Vellore Vellore India

**Keywords:** amyloidosis, bulbar palsy, cranial polyneuropathy, plasma cell dyscrasia

## Abstract

Amyloidosis, a disease with extracellular tissue deposition of fibrils, results in clinical manifestations based on deposition of these fibrils in multiple organ systems. Usual manifestations include nephrotic‐range proteinuria, cardiac failure, hepatosplenomegaly, and skin manifestations. Common neurological manifestations include peripheral and autonomic neuropathies. Cranial neuropathy has been seldom reported and is an unusual clinical feature of amyloidosis. Here, we report an older man who presented with cranial nerve palsies along with other clinical features, including heart failure, proteinuria, weight loss, anorexia and distal symmetric polyneuropathy and was diagnosed with immunoglobulin light‐chain (AL) amyloidosis.

## 1 INTRODUCTION

“Amyloidosis” refers to the extracellular tissue deposition of fibrils composed of low molecular weight subunits of a variety of normal serum proteins.^1^ The four most common causes of systemic amyloid deposition are immunoglobulin light‐chain (AL) amyloidosis, serum amyloid A protein (AA) amyloidosis, hereditary (familial) amyloidosis, and age‐related (senile) amyloidosis.

AL amyloidosis, often associated with an underlying plasma cell dyscrasia, including multiple myeloma, monoclonal gammopathy of unknown significance, Waldenstrom’s macroglobulinemia, and other malignant disorders of plasma cells or lymphoplasmacytic cells must be differentiated from other forms of amyloidosis (eg, AA amyloidosis, hereditary amyloidosis, and senile amyloidosis) as the latter are non‐neoplastic, and can be harmed by an erroneous diagnosis as they do not respond to chemotherapy.^2^

AL amyloidosis can present with protean clinical symptoms and signs that have been well described in the literature.^3^ These include renal, gastrointestinal, cardiac, and neurological manifestations. Neuropathies including mixed sensory and motor peripheral neuropathy (20%) and/or autonomic neuropathy (15%) are common in AL amyloidosis.^4^, ^5^ However, cranial neuropathy has been reported very rarely in AL amyloidosis.^6^, ^7^

We describe the case details of a 62‐year‐old man who presented with predominant bulbar symptoms and cranial neuropathy in addition to the well‐known manifestations and was diagnosed as AL amyloidosis.

## 2 CASE REPORT

A 62‐year‐old man from Bangladesh presented to our tertiary care teaching hospital in southern India on 18 July 2017 with a history of progressive difficulty in swallowing both solids and liquids with intermittent episodes of post‐prandial cough, nasal regurgitation of food, and hoarseness of voice for the past 4 months. There was history of altered bowel habits with loose stools and multiple episodes of melaena for the past 3 months.

He had walking difficulty with paresthesia and numbness of the lower limbs for 6 months. He had significant weight loss (12 kgs) and loss of appetite for 1 year. He was a known case of type 2 diabetes mellitus and essential hypertension on regular drugs. He was a reformed cigarette smoker (20 pack‐years).

In February 2017, he had required admission for congestive heart failure and anemia requiring blood transfusion.

He denied history of fever, rash, joint pains, bone pains, night sweats, jaundice, abdominal distension, headache, seizures, vomiting, blurring of vision, bowel or bladder incontinence or motor coordination problems.

On examination, pulse (96/min, regular) and respiratory rate (20/min) were normal. He had generalized muscle wasting, orthostatic hypotension (BP 90/60 mm Hg supine and 66/50 mm Hg after standing for 3 min), clubbing and macroglossia with a near complete loss of papillae. Neurological examination revealed very weak gag reflex bilaterally (negligible elevation of the soft palate, absent contraction of pharyngeal muscles), very weak palatal and shoulder movements (could not elevate shoulder against minimal resistance), absent ankle jerks with reduced pain, and temperature sensation over the soles and dorsa of both feet. Other general and systemic examinations were within normal limits. Muscle power was 5/5 in the upper and lower limbs.

The differentials considered were gastrointestinal malignancy, hematological malignancies (eg, lymphoma), plasma cell disorders, and gastrointestinal malabsorption syndrome.

Initial investigations revealed a normocytic anemia with hypoproteinemia and hypoalbuminemia, with subnephrotic range proteinuria (Table 1).

**TABLE 1:** Laboratory investigations

Investigation	Result (normal range)
Total white blood cell count (cells/mm^3^)	11 700 (4000‐11 000)^a^
Hemoglobin (g/dL)	11.7 ( >13)^a^
Mean corpuscular volume (fL)	92 (80‐100)
Red cell distribution width (%)	16 (11.5‐14.5)^a^
Iron (µg/dL)	37 (60‐160)^a^
Total iron binding capacity (µg/dL)	155 (300‐400)^a^
Ferritin (ng/mL)	344 (20‐320)^a^
Platelet count (cells/mm^3^)	253 000 (150 000‐450 000)
Sodium (mmol/L)	134 (135‐145)^a^
Potassium (mmol/L)	4.2 (3.5‐5)
Bicarbonate (mmol/L)	26 (22‐29)
Calcium (mg/dL)	8.5 (8.3‐10.4)
Phosphorus (mg/dL)	3.9 (2.5‐4.6)
Creatinine (mg/dL)	0.78 (0.7‐1.4)
Estimated glomerular filtration rate	57 mL/min
Lactate dehydrogenase (U/L)	442 (225‐460)
24‐hour urine protein (mg)	1100 (<150)^a^
Blood‐borne virus screen (HIV, HBsAg, HCV)	Negative
Total bilirubin (mg/dL)	0.2 (0.5‐1)^a^
Direct bilirubin (mg/dL)	0.1 (0.1‐0.5)
Total protein (g/dL)	4.3 (6‐8.5)^a^
Albumin (g/dL)	2.3 (3.5‐5)^a^
Aspartate transaminase (U/L)	14 (8‐40)
Alanine transaminase (U/L)	11 (5‐35)
Alkaline phosphatase (U/L)	51 (40‐125)
C‐reactive protein (mg/L)	24 (<6)^a^
Prothrombin time (s)	11.3 (10‐12.5)
Activated partial thromboplastin time (s)	25.3 (23.5‐33.5)

^a^ Abnormal results

Serum D xylose (0.21 mg/dL) was low (normal > 0.55 mg/dL), and stool was positive for occult blood. Anti‐tissue transglutaminase antibody for coeliac sprue was negative and stool for fat globules was negative.

Serum electrophoresis revealed an overlapping band in alpha 2 region with an increase in kappa free light‐chains and increased kappa/lambda ratio (kappa:1003, lambda: 27.4, K/L ratio: 36.6). Immunofixation electrophoresis was normal. Urine immunofixation electrophoresis was not performed. Skeletal survey did not reveal bone lytic lesions and serum calcium levels were normal. Ultrasound of the abdomen showed starry sky appearance of the liver suggestive of an infiltrative disorder. Echocardiogram showed left ventricular hypertrophy, mild left ventricular systolic dysfunction (ejection fraction 48%) and regional wall motion abnormality in the basal inferoseptal and inferior regions.

Bone marrow examination revealed a normocellular marrow with focal plasmacytosis (20%) and kappa light‐chain restriction on immunohistochemistry consistent with a plasma cell dyscrasia.

Nerve conduction studies (Tables 2, 3, 4) and electromyography (Table 2, 3, 4) showed denervation in the bulbar and cervical myotomes and sensorimotor axonal polyneuropathy with absent sympathetic skin response in lower limbs (suggestive of autonomic small fiber neuropathy). Nasopharyngolaryngoscopy showed mobile vocal cords but pooling of secretions in bilateral pyriform sinuses.

**TABLE 2:** Motor nerve conduction

	Latency	Amplitude	Conduction velocity	Duration	Amplitude ratio	Area VQN
Median right						
Wrist	3.7 ms	5.3 mV	54 m/s	5.7 ms	87.9%	12.6 mVms
Elbow	8.5 ms	4.6 mV	m/s	6.0 ms	102.2%	12.3 mVms
Axilla	10.9 ms	4.7 mV	m/s	6.7 ms	98.3%	15.4 mVms
Erb’s point	15.3 ms	4.7 mV	m/s	6.1 ms	%	12.0 mVms
Median left						
Wrist	3.5 ms	7.2 mV	59 m/s	5.2 ms	97.2%	15.5 mVms
Elbow	7.9 ms	7.0 mV	m/s	5.0 ms	86.0%	14.9 mVms
Axilla	10.0 ms	6.0 mV	m/s	5.5 ms	90.5%	18.4 mVms
Erb’s point	13.8 ms	5.5 mV	m/s	5.4 ms	%	16.9 mVms
Ulnar right						
Wrist	3.1 ms	5.7 mV	45 m/s	6.6 ms	87.1%	13.3 mVms
Above elbow	9.6 ms	4.9 mV	m/s	6.5 ms	%	12.8 mVms
Ulnar left						
Wrist	3.0 ms	4.6 mV	53 m/s	6.5 ms	94.6%	11.8 mVms
Above elbow	8.5 ms	4.4 mV	m/s	6.9 ms	%	10.5 mVms
Peroneal right						
Ankle	3.9 ms	1.8 mV	m/s	6.0 ms	%	5.2 mVms
Fibula (head)	14.5 ms	1.4 mV	36 m/s	ms	74.6%	mVms
Peroneal left						
Ankle	4.4 ms	1.2 mV	m/s	5.8 ms	%	3.3 mVms
Fibula (head)	14.2 ms	1.1 mV	39 m/s	6.4 ms	85.6%	2.8 mVms
Tibial right						
Ankle	4.4 ms	3.5 mV	m/s	5.4 ms	%	5.9 mVms
Popliteal fossa	13.6 ms	2.4 mV	42 m/s	8.0 ms	67.2%	5.7 mVms
Tibial left						
Ankle	4.1 ms	3.9 mV	m/s	6.1 ms	%	12.7 mVms
Popliteal fossa	15.2 ms	3.6 mV	34 m/s	6.7 ms	90.7%	9.5 mVms
Recording on tibialis anterior						
Right	3.4 ms	5.4 mV	m/s	ms	%	mVms
Left	3.7 ms	5.0 mV	m/s	17.1 ms	92.6%	19.0 mVms
Phrenic nerve						
Right	8.5 ms	0.7 mV	m/s	ms	%	mVms
Left	8.5 ms	0.8 mV	m/s	ms	%	mVms

Abbreviations: m/s, meters/second; mm, millimeters; ms, milliseconds; mV, millivolt; VQN, Viking Quest New

**TABLE 3:** Sensory nerve conduction

	Distal latency	Amplitude	Conduction velocity	Segment	Latency difference	Distance VQN
Median right						
Wrist	2.3 ms	24 μV	57 m/s	Index finger‐Wrist	2.3 ms	130 mm
Median left						
Wrist	2.3 ms	21 μV	57 m/s	Index finger‐Wrist	2.3 ms	130 mm
Ulnar right						
Wrist	1.9 ms	21 μV	58 m/s	Little finger‐Wrist	1.9 ms	110 mm
Ulnar left						
Wrist	1.6 ms	20 μV	69 m/s	Little finger‐Wrist	1.6 ms	110 mm
Sural right						
Lower leg	2.1 ms	3 μV	52 m/s	Ankle‐Lower leg	2.1 ms	110 mm
Sural left						
Lower leg	2.1 ms	2 μV	52 m/s	Ankle‐Lower leg	2.1 ms	110 mm
Superficial peroneal right						
Ankle	2.0 ms	9 μV	54 m/s	Dorsum of foot‐Ankle	2.0 ms	110 mm
Superficial peroneal left						
Ankle	2.3 ms	2 μV	52 m/s	Dorsum of foot‐Ankle	2.3 ms	120 mm

Abbreviation: µV, microvolts; VQN, Viking Quest New

**TABLE 4:** Electromyography findings

Muscle	Insertional activity	Spontaneous activity					Interference pattern	Motor units		
		Fibs	Fasc	Myot	PsMyo	+wave		AMPmv	DURms	Remarks
RT tibialis anterior	N		—	—	—	—	N	0.9	7.4	N
RT gastrocnemius	N	—	—	—	—	—	N	0.4	6.3	N
RT vastus lateralis	N	—	—	—	—	—	N	0.8	11.5	N
Rectus abdominis	N	—	—	—	—	—				N
RT FDI	↑	++	++	—	—		↓	0.5	12.7	N
RT APB	↑	+	—	—	—	++	↑ Mildly	1.5	8.2	N
RT EDC	N	—	—	—	—		N	0.8	8.3	N
RT biceps	N	—	—	—	—	—	N	0.6	7.2	N
Tongue	N	—	—	—	—	—				

Abbreviations: AMP, amplitude; APB, abductor pollicis brevis; DUR, duration; EDC, extensor digitorum communis; Fasc, fasciculations; FDI, first dorsal interosseus; Fibs, fibrillations; Myot, myotonia; N, normal; PsMyo, pseudomyotonia; RT, right

Gastrointestinal endoscopy revealed an esophageal ulcer, esophagitis and antral nodule with a smooth duodenal mucosal bulge. Biopsies from both the gastric antrum and the duodenum revealed abundant extracellular and perivascular acellular eosinophilic amorphous deposits of amyloid (showing apple green birefringence on congo red stain and yellowish green fluorescence of thioflavin T). A tongue biopsy was also consistent with amyloidosis. Immunohistochemical typing of the duodenal, gastric and tongue biopsies for AA was negative.

Positron‐emission tomography (PET) computed tomography (CT) whole‐body scan did not reveal any malignancy but showed features of aspiration in both the lungs. Although PET‐CT whole‐body scan has its limitations in diagnosing parenchymal brain lesions, it did not reveal gliosis, infarcts, or other skull‐base lesions. MRI of the brain, though planned, could not be performed. Soon after the procedure, he was found to be unresponsive and died despite resuscitative measures. Cardiac arrythmia was presumed to be the cause of death. The relatives did not provide consent for postmortem examination.

## 3 DISCUSSION

In view of multisystem involvement with peripheral sensorimotor axonal neuropathy, autonomic neuropathy, proteinuria, gastrointestinal involvement, probable liver infiltration, bone marrow showing 20% plasma cells and biopsies from the stomach, duodenum and tongue positive for amyloidosis, we diagnosed our patient as having plasma cell dyscrasia with AL amyloidosis. The striking features were cranial neuropathy (involvement of 9th, 10th, and 11th cranial nerves) with bulbar weakness and significant aspiration on imaging. We felt that cranial neuropathy was the cause of dysphagia, weak palatal and shoulder movements and could not be explained by macroglossia alone.

Although multiple upper limb mononeuropathies, mononeuropathy multiplex, lumbosacral radiculoplexopathy and chronic inflammatory demyelinating polyneuropathy have been reported with AL amyloidosis,^8^, ^9^ association with cranial neuropathy is extremely rare. We found one case series with six cases^7^ and seven isolated cases of AL amyloidosis with cranial nerve palsies reported to date.^6^, ^10^, ^11^, ^12^, ^13^, ^14^, ^15^

Little et al^6^ reported a case of non‐familial amyloidosis due to an IgG kappa‐type plasma cell dyscrasia with multiple bilateral cranial nerve deficits. Traynor et al^7^ reported six patients with cranial neuropathy as a major manifestation of primary amyloidosis. Three patients had multiple cranial nerve palsies. Renal involvement manifested by proteinuria was observed in all of the patients.

Ikeda et al^16^ reported a case of a 72‐year‐old man with Japanese familial amyloidosis (FAP I) presenting with bulbar palsy. We did not perform genetic testing on our patient as we felt a genetic cause was unlikely.

The exact pathophysiology of neuropathy in amyloidosis is not clear. Possible mechanisms include endoneurial amyloid deposition.^17^ Other clues would include non‐diabetic nephrotic‐range proteinuria, restrictive cardiomyopathy or otherwise unexplained congestive heart failure, hepatosplenomegaly, carpal tunnel syndrome, unexplained facial or neck purpura, malabsorption or macroglossia.^18^

To conclude, cranial neuropathy is an extremely rare presentation of AL amyloidosis and, exceptionally, the only clinical feature of this disease. AL amyloidosis should be suspected in patients presenting with cranial neuropathy and plasma cell dyscrasia along with other typical manifestations of AL amyloidosis, such as restrictive cardiomyopathy, non‐diabetic proteinuria, purpura, malabsorption, and macroglossia. A high index of suspicion is required to diagnose the disease accurately and treat it promptly.

## CONFLICTS OF INTEREST

Nothing to disclose.

## AUTHOR CONTRIBUTIONS

Writing of paper: All authors. Data collection and literature review: Dr Abhinav agarwal. Design and literature review: Dr Gopinath Kango Gopal.
